# Identifying the causes and consequences of pregnancy in Iranian Kurdish women under the age of 18: A grounded theory study

**DOI:** 10.1016/j.heliyon.2025.e42271

**Published:** 2025-01-24

**Authors:** Javad Yoosefi lebni, Ahmad Ahmadi, Seyed Fahim Irandoost, Mandana Saki, Hossein Safari, Nafiul Mehedi

**Affiliations:** a Social Determinants of Health Research Center, Lorestan University of Medical Sciences, Khorramabad, Iran; b Faculty of Psychology and Educational Sciences, Allameh Tabataba'i University, Tehran, Iran; c Department of Community Medicine, School of Medicine, Urmia University of Medical Sciences, Urmia, Iran; d Health Promotion Research Center, Iran University of Medical Sciences, Tehran, Iran; e School of Health and Related Research (ScHARR), The University of Sheffield, Sheffield, S10 2TN, UK

**Keywords:** Adolescents, Child marriage, Pregnancy, Women, Grounded theory

## Abstract

**Background:**

Since teenage pregnancy is one of the major challenges for healthcare systems worldwide and can pose risks to the health of young mothers and their infants, the present study was conducted to identify the Identifying the causes and consequences of pregnancy in Iranian Kurdish women under the age of 18.

**Method:**

The present qualitative research was conducted using the grounded theory method among women with experience of pregnancy under the age of 18 and key informants who had experience and knowledge in this field. Data were collected through semi-structured face-to-face interviews with 26 women and 17 key informants who were selected through purposive, snowball, and theoretical sampling methods. Sampling continued until theoretical saturation. The data were collected and analyzed for 8 months, from November 2021 to June 2022. Data analysis was performed based on the approach of Strauss and Corbin in the MAXQDA-2018 software environment. The Guba and Lincoln criteria were observed to ensure the trustworthiness of the data and results.

**Results:**

After the data analysis and coding process, the conceptual model of causes and consequences of pregnancy in adolescents emerged, including 1) predisposing conditions (*sociocultural factors*: Social learning, misconceptions about fertility and childbirth, preventing stigma), 2) causal conditions (*individual factors*: lack of knowledge on how to prevent pregnancy, improper use of contraceptives, inadequate knowledge about the risks of pregnancy in adolescence, fear of the side effects of using contraceptives, filling the vacuum of loneliness, *family factors*: husband's and his family's pressure, committing her husband to life, consolidating her position in family), 3) intervening conditions (s*tructural factors*: no barriers to pregnancy, difficult access to contraceptives), 4) strategies and interactions (*positive reactions*: trying to prepare herself for raising a child, taking better care of herself and her child, *negative reactions*: trying to kill herself and the kid, fear and concealment), and 5) consequences (*destructive consequences*: *threats to the health of the child, threat to mother's health, inadequate access to health services*, *constructive consequences*: increase of support, strengthen the sense of empowerment).

**Conclusion:**

Pregnancy in adolescence is influenced by socio-cultural, family, personal, and structural factors that can lead to positive and negative consequences for women, which in most cases make the lives and health of them and their children difficult. The findings of the study can be used in the areas of health and social policy, program planning, and designing interventions and educational programs aimed at changing beliefs and cultural attitudes related to pregnancy under the age of 18 at the individual, family, and societal levels.

## 1 Introduction

In recent decades, children's rights have become one of the most important issues in the world [1]. One of the examples of violations of children's rights is child marriage [2], which, according to its common definition, refers to marriage under the age of 18 [3,4]. Although child marriage can happen to both men and women and endanger the health of both, it has been discussed more with women because it affects women's lives more and is considered an example of gender discrimination [5]. Child marriage is more common among girls than boys and has more devastating consequences [[6], [7], [8]]. Child marriage statistics vary from country to country, but in general, child marriage is more common in poor countries [3,9].

It has been estimated that between 2010 and 2030, there will be about 130 million early marriages, of which 14 million girls marry each year, and many of them are under 15 years old [10]. According to statistics, the child marriage rate in Iran is 19.6 % in rural areas and 13.7 % in urban areas [11]. Adolescent girls give birth to approximately 16 million babies each year (11 % of births worldwide) [12]. In Africa, 33 % of adolescent girls give birth before the age of 18, and 3.5 % even before the age of 15 [13]. In Asia, Bangladesh, Nepal, and India have reported the highest prevalence of adolescent pregnancy at 35 %, 21 %, and 21 %, respectively [14]. The adolescent pregnancy rate in Iran is about 7 % [15,16].

Early marriage has many social and health consequences [17] that can lead to many challenges in the lives of adolescent girls [18,19]. One of the results of early marriage is pregnancy in adolescence [20,21]. Adolescent pregnancy is a major global problem [2,22,23] and one of the leading causes of death in girls aged 15–19 in developing countries [24,25]. About 11 % of births worldwide are by adolescents aged 15–19 years, and more than 90 % of these births occur in low- and middle-income countries [26]. Early marriage in Iran can lead to physical and psychological challenges such as high-risk pregnancy and childbirth, physical illnesses, regret, and remorse. It can also result in family problems such as experiencing violence, dissatisfaction with marital life, multiple role expectations, and a lack of independence in family life, which can ultimately lead to social consequences such as social deviance, deprivation of social and health services, social isolation, and limited job and educational opportunities [27].

Pregnancy in adolescents is influenced by various factors [28]. In a study in five African countries, more than half of all pregnancies among adolescents are unintended, and factors such as education, age at the time of first sexual intercourse, family wealth, and family structure are important determinants [29]. In another study conducted in Russia, several factors were found to be directly related to adolescent pregnancy. These factors included low maternal education, alcohol use, family structure, low academic grades, and higher frequency of sexual intercourse [30]. In a review study, it was reported that limited education, low socioeconomic status, inadequate access to contraceptive methods, and non-use of them were factors associated with pregnancy among adolescents in low- and middle-income countries. There is also evidence that early marriage, living in a rural area, early sexual initiation, and belonging to an ethnic or religious minority group also increase the risk of pregnancy in adolescents [31]. Pregnancy in adolescence can have risks such as dropout [20], unsafe abortion [16], preterm delivery, low birth weight, growth retardation, stillbirth [32], and maternal death due to pregnancy [33,34]. Mangeli et al. (2017) also reported that the increase in the burden of responsibility, experiencing physical problems, receiving insufficient support, inefficiency in the role of the mother, and emotional and psychological problems have been the most common problems of pregnant adolescents in Iran [35].

Regarding the widespread effects and consequences of pregnancy in adolescence, the need to investigate and identify the causes and consequences of this phenomenon becomes apparent. A review of the literature in this field showed that most studies have a quantitative approach [[36], [37], [38]]. Each culture has its view on marriage and pregnancy, and the model of marriage and pregnancy varies between countries and even within a country among ethnic groups. The results of other studies in other countries and even within Iran between other ethnicities are not generalizable to the study area that is populated by Kurds due to the different social and cultural contexts, thus the need for a qualitative study of this phenomenon becomes obvious [27]. Also, in Iran, a small number of qualitative studies on pregnancy in adolescence have been done, but so far no research has been done in the study area. Because the study population is different in terms of ethnicity, language, and culture from other parts of Iran, it is necessary to study this phenomenon qualitatively and with a grounded theory approach. In other words, early pregnancy is a complex matter that is rooted in cultural and social relations, and the reasons and contexts for its formation are complex, hidden, and not easily visible. Therefore, the discovery of the hidden layers of this phenomenon is not possible except with a qualitative method and grounded theory. Therefore, this study aimed to identify the causes and consequences of pregnancy in Iranian Kurdish women under the age of 18. Due to the relatively high prevalence of child marriage in Kermanshah province and the consequently significant statistics of pregnancy under the age of 18, this study was conducted in this province.

## 2 Methods

### 2.1 Design

Utilizing grounded theory methodology and a qualitative approach, pregnancy during adolescence was examined as a complex and involved phenomenon in order to identify and elucidate its various aspects. This is because qualitative research is deemed the most suitable method for comprehending complex phenomena, as supported by existing literature [39,40]. The quantitative method is deemed unsuitable due to the dispersion and lack of knowledge regarding the statistical population, as stated in reference [27]. Furthermore, a lack of a universally accepted instrument or survey exists to effectively accomplish the aims of this investigation and satisfy the scientific inquisitiveness of the researchers. The use of the qualitative method in this study was primarily driven by the absence of alternative means to address the research inquiries and attain the research objectives, thereby rendering the qualitative method the most viable option. Consequently, the qualitative approach was chosen following extensive preliminary investigations in alignment with the research inquiries and goals. The grounded theory approach was employed because the occurrence of adolescent pregnancy is formed under certain social and cultural conditions, and the grounded theory approach is more suitable for understanding these social and cultural conditions.

### 2.2 Participants

The research sample consisted of Kurdish females residing in Kermanshah province who became pregnant before the age of 18. Additionally, the study included key informants who possessed relevant experience and expertise in this particular area. Considering the familiarity and experience of the research designers in the field of marriage and pregnancy in children under 18 years old and the relative prevalence of this issue among Kurdish women, this group of women was selected for the study. Furthermore, the selection of Kurdish women in Kermanshah province was made in consideration of the presence of other ethnic minorities with distinct cultures and societies, warranting a deeper comprehension of this phenomenon within the Kurdish populace. The study's inclusion criteria for female participants comprised having undergone pregnancy before the age of 18 through formal marriage, being below 22 years of age during the research and pregnancy period, residing in Kermanshah province at the time of the study, being fluent in Kurdish, and expressing a desire to partake in the research. The study's exclusion criteria encompassed individuals who were above 22 years of age, those who did not identify as Kurdish, and those who expressed a lack of willingness to participate in the study. Women were not remunerated for participating in the study, but their travel expenses were paid by the research team. Three of the samples (women) refused to participate in the study and one person was excluded from the study during the interview. To gain a deeper understanding of the phenomenon, interviews were conducted with key informants. The individuals identified as key informants were relied upon by the researcher to provide insight and information that facilitated a deeper understanding of the phenomenon being investigated, including its latent dimensions. The inclusion criteria for key informants encompassed possessing relevant knowledge regarding the causes or ramifications of adolescent pregnancy as well as exhibiting a willingness to engage in research.

### 2.3 Data collection

The sampling process was initiated through a deliberate approach and subsequently progressed through the use of snowball and theoretical sampling techniques [41]. Upon completion of the interview, participants were requested to provide the researcher with introductions to other individuals who satisfied the inclusion criteria as part of the snowball sampling technique. Theoretical sampling involves selecting subsequent participants based on their potential to shed light on the emerging categories following the identification of the initial categories [42]. The researcher commenced the identification of samples by visiting health centers and local trustees to compile a roster of women who had undergone adolescent pregnancy. The interviews were then initiated based on the aforementioned list. The sampling methodology employed in this study persisted until the point of theoretical saturation, whereby the recurrence of additional data and the continuation of interviews failed to contribute novel insights to the research. Overall, theoretical saturation was achieved in this research by conducting interviews with 26 women (as presented in Table 1) and 17 key informants (as shown in Table 2). The scheduling of the interviews was determined by the participants in terms of both timing and location. The majority of female participants were subjected to interviews at healthcare facilities, residential dwellings, and communal areas such as parks. The site of the interview conducted with the key informants was their respective workplaces. In light of the delicate nature of the topic under investigation and to facilitate the participants' candid and open expression of their experiences and emotions, a proficient female interviewer who possessed expertise in conducting semi-structured interviews and held a master's degree in women's studies was engaged. The primary researcher, possessing adequate expertise and proficiency in qualitative research and interviewing, conducted interviews with key informants.

**Table 1:** Demographic information of the participants (n = 26).

**variables**	**Group**	**Frequency (Percentage)**
Education	Illiterate	4 (15.4)
	Under diploma	10 (38.4)
	Diploma	7 (27)
	Higher than diploma	5 (19.2)
Age	15–18	15 (57.7)
	18–22	11 (42.3)
Age of the first pregnancy	Under 15	7 (27)
	15–18	19 (73)
Place of residence	Rural	12 (46.2)
	Urban	10 (38.4)
	Nomadic	4 (15.4)
Occupational status	Housewife	14 (53.8)
	Student	6 (23.1)
	Self-employed	6 (23.1)
Husbands' ages (in year)	Under 25	4 (15.4)
	25–35	12 (46.2)
	Above 35	10 (38.4)

**Table 2:** Demographic information of key informants participating in the study (n = 17).

NO.	Sex	Age	Education	Occupation	Marital status	duration of interview
1	Female	45	Medicine (Specialist)	Obstetrician	Married	50
2	Female	31	BA of midwifery	Midwife	Single	65
3	Male	38	MA of educational management	Headmaster	Married	50
4	Female	71	Illiterate	Traditional midwife	Widowed	40
5	Male	39	MA of laws	Lawyer	Single	45
6	Female	55	Medicine (Specialist)	Obstetrician	Married	50
7	Female	35	MA of midwifery	Midwife	Married	80
8	Male	44	BA of social work	Social worker	Married	60
9	Male	32	PhD of counseling	Family counselor	single	75
10	Female	37	MA of counseling	Family counselor	–	75
11	Female	29	BA of counseling	School counselor	Married	60
12	Male	36	PhD of sociology	University lecturer and researcher in the field of women and children	Married	85
13	Male	30	MA of psychology	Researcher in the field of women and children	Single	40
14	Female	34	MA of studies of women	Researcher in the field of women and children	Married	45
15	Female	48	Illiterate	Mother-in-law and housewife	Married	50
16	Male	54	Illiterate	Father-in-law and farmer	Married	60
17	Female	38	Diploma	Mother and housewife	Married	60

The data collection process was performed using semi-structured interviews; the interviews were conducted face-to-face and individually. In the way that the researcher first presented a brief biography of himself. Then, specialized explanations about the objectives of the research and the process of conducting the research were provided to the participants, and the interview began by asking a few demographic questions. The interview was conducted based on the questions in the interview guide. The interview guide was compiled after reviewing the literature in this field and several brainstorming sessions with the research team members, and it was approved by conducting four pilot interviews (Table 3). Of course, the order of the interview questions was not the same for the participants, and the order of the questions changed based on their answers. It should be noted that the interview question guide for key informants was different from the participating women's question guide and focused more on the causes and consequences of early pregnancy.

**Table 3:** Question guide of the interview with the participating women.

**NO.**	**Questions**
1	What made you get pregnant? Explain.
2	Did you want to get pregnant yourself? If yes, explain why.
3	Did you get pregnant unintentionally? If yes, explain.
4	How familiar were you with contraceptives before becoming pregnant? Explain.
5	Were your family and friends involved in your early pregnancy? If yes, explain.
6	How did you feel when you first found out you were pregnant? And what was your reaction?
7	What problems and challenges did you face during your pregnancy? Explain.
8	How did people around you react to your pregnancy?
9	Before you became pregnant, what were your perceptions and feelings about pregnancy?
10	In general, how do you evaluate pregnancy during adolescence?
11	If you were to go back in time, would you still decide to become pregnant? Why?

### 2.4 Data analysis

Data analysis was performed based on the approach of Strauss and Corbin [39]. The first author and the corresponding author, after the first interview, transcribed the text of the recorded interview word for word on the same day of the interview or the next day, and at the same time the analysis process began and the obtained information was used to prepare the next questions from the interviewees, and this process continued until the end of the interviews. In the data analysis process, three coding steps, including open coding, axial coding, and selective coding, were used. In the open coding step, which is a kind of microscopic analysis of data, the data were broken down to consider all possible meanings. In this step, the text of the interviews was read several times, and the focus of the research team was on constructing concepts from the data. In the second step, which is axial coding, the initial codes and categories that were created in open coding were compared with each other, and while merging similar cases, the categories that were related to each other were placed around a common axis. Careful comparison of codes was continuously carried out during this stage. The researchers then compared each category to another to ensure that the categories were distinct from each other. Then, focusing on the circumstances that led to the phenomenon of pregnancy in adolescence, the contexts in which the phenomenon occurred, and, the strategies used to control the phenomenon were identified through selective coding. In fact, in the selective coding, categories were integrated and refined, and the main model for explaining pregnancy in adolescence was formed (Table 4).

**Table 4:** An example of data analysis.

Categories	Subcategories	Codes	Quotations
Structural factors	No barriers to pregnancy	No job, not continuing education	"I did not study or do anything to delay my pregnancy" "Many teenage women drop out of school when they get married, and most of them become housewives. This condition affects their pregnancy"
	Difficult access to contraceptives	Living in the village and being away from pharmacies, the high cost of contraceptives, the limited supply of contraceptives, the taboo of buying contraceptives	"I wanted to get pregnant a few years after marriage, but we were in the village and I could not go to the city and buy contraceptives" "My husband was a worker. If I wanted to get contraceptives, our costs would go up a lot, so we used natural contraceptive methods, and that made me pregnant"

### 2.5 Trustworthiness

The Guba and Lincoln criteria were observed to ensure the Trustworthiness of the research [43]. To increase dependability, all research colleagues were informed about analysis and coding, and in the meetings that were held, they expressed their opinions. Finally, the names of the categories and subcategories were finalized with the approval of all authors. Credibility was achieved through the researcher's prolonged engagement with the research field and his contact with the participants, which on the one hand helps to gain the participants' trust and on the other hand helps the researcher to understand their experiences. Data coding and analysis were also sent to six participating women and eight key informants to confirm whether the results expressed their views and opinions or not; the results were approved by them. On the other hand, researchers tried to make the participants as diverse as possible in terms of demographic characteristics to get more comprehensive information. Conformability was obtained through the researcher's neutrality and agreement on codes and categories with five experts familiar with pregnancy in adolescence and qualitative research. To increase the transferability of the research, a complete description of the whole research process was provided for each of the subcategories and the code, and many direct quotations were given. Also, a table of categories, subcategories, codes, and the whole section of findings was sent to five women who met the inclusion criteria but did not participate in the study to assess whether the findings of the study were by their experiences and feelings or not; they all approved it.

### 2.6 Ethical considerations

Ethical considerations of the research included receiving the code of ethics from the Health Promotion Research Center of Iran University of Medical Sciences with the code (IR.IUMS.REC.1400.1215), coordinating and obtaining permission to enter the research environment, explaining the purpose of the research and the interview method, assuring participants that their personal information, such as their name and address, would be kept confidential, gaining informed consent to participate in research and record conversations, as well as the right to withdraw from research at any time. Also, in the case of women under the age of 18, written consent was obtained from their husbands to prevent any sensitization and tension in the family.

## 3 Results

**Fig. 1:**
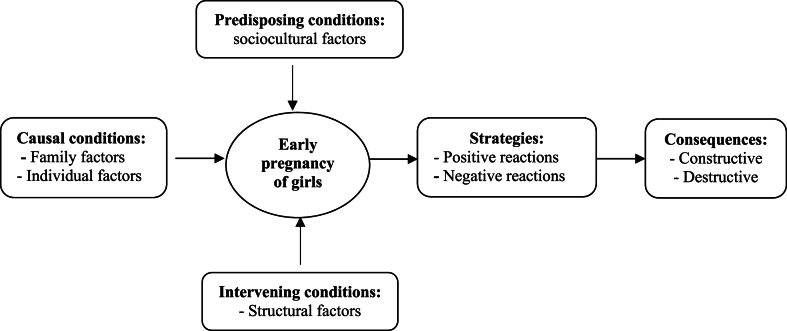
Conceptual model of causes and consequences of adolescent pregnancy in Kermanshah, Iran Identifying the causes and consequences of pregnancy in adolescents based on a grounded theory study.

26 women and 17 key informants participated in this study. From the data analysis, 8 categories, 22 subcategories, and 106 primary codes were obtained (Table 5), and after analyzing the data, a conceptual model of pregnancy causes and consequences in adolescents was drawn (Fig. 1).

**Table 5:** The codes, subcategories, and categories obtained from the analysis of interviews.

Paradigm structure	Categories	subcategories	Codes
Predisposing conditions	Sociocultural factors	Social learning	Experience of pregnancy in adolescence in the family, the experience of pregnancy in adolescence among friends, the experience of pregnancy in adolescence among relatives
		Misconceptions about fertility and childbirth	The sooner you get pregnant, the healthier your baby will be. The best time to get pregnant is the first year of marriage. If the pregnancy is delayed, the couple will not have the patience to have children. Pregnancy in adolescence is good for the health of mother and child, a girl who has menstruation has the conditions of pregnancy, and delaying pregnancy is a sin
		Preventing stigma	Preventing the stigma of being infertile, Preventing the stigma of being lazy, Preventing the stigma of not loving her husband, Preventing the stigma of being irresponsible
Causal conditions	Individual factors	Lack of knowledge on how to prevent pregnancy	Less familiarity with new contraceptives, insufficient familiarity with contraceptive methods
		Improper use of contraceptives	Improper use of birth control pills, improper use of condoms, improper use of other methods of contraception
		Inadequate knowledge about the risks of pregnancy in adolescence	Low knowledge about the conditions and consequences of early pregnancy, little knowledge about the conditions and complications of early childbirth
		Fear of the side effects of using contraceptives	Fear of delayed pregnancy and getting infertile, fear of using contraceptives and getting infertile
		Filling the vacuum of loneliness	Finding a companion, getting entertained and busy
	Family factors	Husband's and his family's pressure	Husband's family pressure to have children, husband's request and desire for pregnancy
		Committing her husband to life	Preventing divorce, increasing the husband's sense of responsibility for life, increasing the husband's attention to her
		Consolidating her position in family	Strengthening her position in the opinion of her husband, strengthening her position in the opinion of her husband's family
Intervening conditions	Structural factors	No barriers to pregnancy	No job, not continuing education
		Difficult access to contraceptives	Living in the village and being away from pharmacies, high cost of contraceptives, limited supply of contraceptives, taboo of buying contraceptives
Strategies and interactions	Positive reactions	Trying to prepare herself for raising a child	Increasing information about pregnancy, increasing information about childbirth, increasing information about raising children, increasing information about nutrition and behavior during pregnancy, practicing how to change diapers
		Taking better care of herself and her child	Following the protocols of pregnancy, paying more attention to her health and the child, performing screenings, and following the instructions of health experts and doctors
	Negative reactions	Trying to kill herself and the kid	Attempted abortion, abortion, suicide attempt
		Fear and concealment	Denial of pregnancy, fear, hiding pregnancy from husband, hiding it from families, hiding it from health centers
Consequences	Destructive consequences	Threats to the health of the child	Stillbirth, death of newborn baby, low birth weight, congenital anomalies, high risk of sudden infant death syndrome (SIDS), risk of mental retardation, brain injury and birth injuries
		Threat to mother's health	Malnutrition, Frequent hospitalization during pregnancy, Excessive bleeding, physical weakness, Hard delivery, Cesarean section
		Inadequate access to health services	Knowing late about pregnancy, not receiving adequate counseling, and not receiving proper health services
	Constructive consequences	Increase of support	Increase of information support, an increase of psychological support, an increase of support related to pregnancy, childbirth, and childbearing
		Strengthen the sense of empowerment	Increased self-confidence, increased self-esteem, increased sense of self-efficacy

### 3.1 Predisposing conditions

Predisposing conditions for pregnancy in adolescence emerged in the form of social and cultural factors.

#### 3.1.1 Sociocultural factors

**Social learning**. Due to the high rate of pregnancy in adolescents in the study area, this feature is modeled as a visible behavior of others. Most of the participants stated that among family members, friends, and relatives, there are people who have experienced pregnancy in adolescence. Adolescent pregnancy is a common behavior in the study areas, and this issue leads to imitation and modeling by other young women.

"We are three sisters; all of us became pregnant under the age of 18. I was the youngest of them all. When I saw my sisters get pregnant at a young age, I said to myself, there was no problem for me." (21-year-old woman, under diploma)

"Most of my friends who get married early will have children soon. I am like all of them." (18-year-old woman, under diploma)

"There are many in the family who, like me, have experienced pregnancy in adolescence." (22-year-old woman, higher than diploma)

"Some teenage women learn from their peers or their mothers to give birth early (32-year-old man, family counselor)

**Misconceptions about pregnancy and childbirth**. There was a set of misconceptions about fertility and childbirth in the study area that led young girls to try to get pregnant as soon as they got married. Most of these beliefs have been inherited from the distant past and have spread among the youth. Some of the participants believed that women who had children earlier had healthier children and that the best time to get pregnant was in the first year of marriage. Some also considered menstruation a criterion for having children and believed that teenage pregnancy was good for the baby and the mother. Other young women believed that using contraceptives and delaying pregnancy were sins and tried to get pregnant sooner.

"The sooner a woman becomes pregnant, the healthier the baby she gives birth to." (48-year-old illiterate mother-in-law)

"Some of my clients say that we thought that if we delayed the pregnancy, our children might have problems later, or we would not be in the mood for kids." (31-year-old midwife with a bachelor's degree)

"My mom always said that the sooner you get pregnant, the better for you and your baby." (16-year-old woman, under diploma)

"When a girl experiences menstruation, it means she can get pregnant; she should not delay pregnancy." (48-year-old illiterate mother-in-law)

"It is a sin to delay pregnancy, and God may be upset and not give you a child at all." (38-year-old mother with a diploma)

**Prevention of stigma.** Due to the prevalence of pregnancy among young women in the region, women who delay pregnancy will be stigmatized as infertile by families and the community, and since childbearing is associated with many responsibilities, women who delay pregnancy are considered lazy women who do not dare to take responsibility for raising a child. Therefore, some participants became pregnant to avoid this type of stigma.

"I encouraged my daughter to have a child in the first year of marriage so that her husband's family does not say she is infertile." 38-year-old mother with a diploma

"I wanted to have a baby soon and let everyone know that I'm not lazy and I can do my job." (21-year-old woman with a diploma)

"Here, if you have children late, everyone thinks that you are not satisfied with your life and that you do not love your husband. That is why I tried to have a child soon." (17-year-old woman, higher than diploma)

"Because early pregnancy is an advantage here, many women who come to me get pregnant to show others that they have a lot of responsibility and are no longer children." (55-year-old obstetrician)

### 3.2 Causal conditions

In this study, causal conditions consisted of two categories: individual factors and family factors.

#### 3.2.1 Individual factors

Part of this category refers to the lack of knowledge on how to prevent pregnancy, and another part refers to the improper use of contraceptives. Also, women are not well aware of the dangers of pregnancy in adolescence; the fear of getting infertile and filling the vacuum of loneliness are other sub-categories that lead to pregnancy in adolescence.

**Lack of knowledge on how to prevent pregnancy**. Most women with early marriage experience have a low level of education and limited access to the Internet for awareness. Therefore, they are not familiar with how to prevent pregnancy and do not know the means of contraception or even ways to prevent it. Of course, most of this issue goes back to families and the country's education system, because families never teach their daughters about these issues due to the taboo of sexual issues, and the country's education system has no lessons, units, or any training on ways to prevent pregnancy, etc. in its schedule. Hence, teenage girls have no suitable source to receive such instructions.

"I did not want to get pregnant so soon, but I did not know how to prevent it; I thought it was more related to my husband than myself." 15-year-old woman, under diploma

"I did not know at all how and by what means it could be prevented; no one had told me." (19-year-old woman with a diploma)

"Many pregnant women who come to me have unwanted pregnancies. They do not know anything about contraceptives. Even their families do not tell them anything." (45-year-old obstetrician)

"When my daughter got married, I could not tell her anything about contraceptives. We had never talked about such things. I thought it would be disrespectful for me to talk about them. (38-year-old woman with a diploma)

**Improper use of contraceptives**. Regarding the taboo and lack of adequate instruction, even if they know about the available ways of contraception, due to insufficient literacy about how to use these contraceptives, pregnancy will happen. Some of the participating women, despite having access to contraceptives, could not prevent pregnancy due to not receiving training on how to use these devices and had an unintended pregnancy.

"I did not want to get pregnant; I was taking pills, but I do not know why I got pregnant." (16-year-old woman, under diploma)

"I thought I should only take the pill after having sex, so I got pregnant without wanting to." (Illiterate 20-year-old woman)

"My husband used condoms, but I got pregnant. I do not know; maybe we did not know how to use it." (22-year-old woman, higher than diploma)

"Many teenage women do not know the right way to use contraceptives; when they find out they are pregnant, they get really surprised. But when I ask them about how to prevent pregnancy, I see that they didn't do it right at all." (35-year-old midwife with an MA degree)

**Inadequate knowledge about the risks of pregnancy in adolescence.** Many adolescent women have inadequate knowledge about adolescent pregnancy issues before pregnancy, and if they are familiar with the conditions of pregnancy and its dangers, they will be less encouraged to become pregnant, but such knowledge and education have not been provided for them before.

"I did not know at all that pregnancy as a teenager could endanger my health and the health of my child if I knew I would never get pregnant so soon." (18-year-old woman under diploma)

"Nobody told me about the conditions of childbirth and pregnancy, and I did not know at all what awaited me when I became pregnant." (17-year-old woman, under diploma)

"Many women and even their husbands do not know anything at all about the dangers of pregnancy in adolescence because no training or information has been provided in this field." (30-year-old man, researcher in the field of women and children)

**Fear of the side effects of using contraceptives**. Most women who experienced adolescent pregnancy thought that if they delayed pregnancy or used contraceptives, they might become permanently infertile and not be able to experience pregnancy. Hence, they avoided delaying pregnancy. Many women in the area have a negative view of contraceptives and think that these contraceptives are dangerous to their health and may prevent them from getting pregnant again in the future.

"I've heard a lot that those who delay pregnancy may not get pregnant anymore, so I got pregnant early." 20-year-old woman with a bachelor's degree.

"My mother always said that these contraceptives were dangerous, and if they were used too much, it would make a woman never get pregnant again." (22-year-old woman, illiterate)

"There has long been a negative view of contraceptives, and it has been believed that the use of contraceptives endangers women's health in the future." (34-year-old woman, MSc in Women Studies)

**Filling the vacuum of loneliness**. Some participants became pregnant due to loneliness and a lack of companionship and considered childbearing a form of entertainment to fill their lonely time. This was more common among women whose husbands lived far away.

"My husband had to go to work in Tehran. I was very lonely, and I was very annoyed. I told myself to bring a child so that I would not be alone and have fun." (21-year-old woman with a diploma)

"For me, who was always alone, a child was a blessing. At that time, my husband used to go out in the morning and come back at night. My father's house and my father-in-law's house were not close enough for me to visit so that I did not get bored, so I decided to have a child." 20-year-old woman with a diploma.

"Before I had a baby, I was very lonely, but after that, I really did not feel lonely anymore, and I was entertaining myself with the baby." (21-year-old woman with a diploma)

#### 3.2.2 Family factors

This category refers to the factors that encouraged or forced a person to become pregnant within the family. This category included the sub-categories of family and husband pressure, committing the husband to life, and consolidating her position in the family.

**Husband and his family's pressure**. Some participants stated that the main reason for pregnancy was the pressure exerted by their husbands or their families on them to have an early pregnancy. In fact, the study area has shown that many men and their families tend to experience early pregnancy as a natural event, leading to earlier pregnancies.

"When we got married, I told my husband that I would not have children until a few years later." He said, sure. "No problem.' But a few months after the marriage, I saw that his mother was telling him, why doesn't your wife have children?" (18-year-old woman, under diploma)

"I had no interest in having children, but at the insistence of my husband and sisters-in-law, I had children." (21-year-old woman with a diploma)

"Many families here think that the sooner their bride has a baby, the more successful she is, which is why many teenage girls who get married experience pregnancy very soon." (37-year-old woman, family counselor)

**Committing the husband to life**. In Iranian society, there is a presumption that having a child makes a man feel more committed and responsible to life than before. This is why some teenage women get pregnant to make their men commit to life or get more attention from them. Also, because having children often makes men reluctant to divorce and separate from their wives, some women use pregnancy as a strategy to prevent divorce.

"My relationship with my husband and his family was not very good. I was very afraid that he would divorce me, so I told myself to have a child so that he could not divorce me." (22-year-old woman with a diploma)

"My husband was always with his friends and did not think about our life at all, so I said that if we have children, he might change his behavior." (17-year-old woman, under diploma)

"I wanted my husband to pay more attention to me. I tried different ways, but it only took a few days, and then she lost attention again. I told myself that maybe pregnancy would make him pay more attention to me forever." (20-year-old woman with a diploma)

"Some families and even women themselves think that by having children, they can better attract their husbands and put them on the path of life. Therefore, especially in lives where there is chaos and tension, pregnancy occurs earlier, and most of these lives after pregnancy not only do not get better but get worse. (44-year-old man, social worker)

**Consolidating her position in the family**. According to the traditional context of the Kurdish regions of Iran, having a child is a great advantage for a woman because she has caused her husband's generation to continue. In such a situation, if the child is a boy, he will be given more attention by her husband and his family. Of course, in the last two or three decades, this situation has changed, and families have found a more balanced view of the daughter. Of course, traces of this discriminatory view may still be seen in rural and traditional areas.

"I knew that if I had a child, I would raise my position with my husband and his family; because my child was a boy, my position became even better." (17-year-old woman, under diploma)

"Before I got pregnant, my husband's family did not accept me much, but after finding out I was pregnant, they paid a lot of attention to me." (22-year-old woman with a diploma)

### 3.3 Intervening conditions

Intervening conditions refer to conditions that accelerate pregnancy in adolescence and are included in the category of structural factors.

#### 3.3.1 Structural factors

This category consists of two subcategories: lack of barriers to pregnancy and difficult access to contraceptives that advance the pregnancy process of adolescent women.

**There are no barriers to pregnancy**. Most women in Kurdish areas drop out of school due to social and cultural conditions, and because working conditions are not available for them and they have to stay at home, there is no longer a barrier to their pregnancy because continuing education and working conditions can delay women's pregnancies.

"I did not study or do anything to delay my pregnancy." (16-year-old woman, under diploma)

"Many teenage women drop out of school when they get married, and most of them become housewives. This condition affects their pregnancy. (36-year-old man, PhD in Sociology)

**Difficult access to contraceptives**. Most of the women studied lived in rural areas and did not have much access to pharmacies to buy contraceptives. Also, the high price of contraceptives can be effective because most women are from poor or middle-class families and find it expensive to buy contraceptives. In addition, in Iran, contraceptives are only available in pharmacies, which makes them difficult for women to access. These drugs are less available in hospitals and public pharmacies, and because of the taboos that exist, women are usually embarrassed to buy these drugs. Another major reason is the taboo of talking about contraceptives and buying them, which makes it very difficult and even impossible for women to buy contraceptives.

"I wanted to get pregnant a few years after marriage, but we were in the village, and I could not go to the city and buy contraceptives." 17-year-old village woman, under diploma

"My husband was a worker. If I wanted to get contraceptives, our costs would go up a lot, so we used natural contraceptive methods, and that made me pregnant." (20-year-old woman with a diploma)

"Only pharmacies offer contraceptives, but most of them do not have them." (30-year-old man, researcher in the field of women and children)

"I'm really ashamed to go to the pharmacy and say I want a condom; it's too hard for me." (16-year-old woman, under diploma)

"Many women and even their husbands are ashamed to buy condoms because buying condoms is taboo in Iran, especially in sparsely populated urban areas." (37-year-old woman, family counselor)

### 3.4 Strategies and interactions

Strategies and interactions deal with how pregnant women react to and interact with pregnancy; two categories of negative and positive reactions were identified. In the positive reactions of pregnant women, they had a positive view of pregnancy and by raising their awareness in the field of pregnancy, they prepared themselves for it so that they could better take care of themselves and the baby. But in other cases, pregnant women reacted negatively to pregnancy and did not consider themselves ready to take responsibility for pregnancy, so their pregnancy was accompanied by fear and they tried to destroy the child or hide their pregnancy from others.

#### 3.4.1 Positive reactions

This category deals with the positive reactions of women to the awareness of their pregnancy and includes two subcategories: trying to prepare oneself for raising a child and taking better care of oneself and the child.

**Trying to prepare herself for raising a child**. Some adolescent women reacted positively after becoming aware of their pregnancy, and in the first step, they tried to increase their knowledge about pregnancy, childbirth, childbearing, nutrition, and how to behave during pregnancy. In some cases, teenage women practiced changing diapers as well as other babysitting behaviors.

"When I found out I was pregnant, I was stressed at first, but I soon came to terms with it and started reading about pregnancy, childbirth, etc." (22-year-old woman, higher than diploma)

"I could cope with my pregnancy very soon, so I increased my knowledge in this field." (18-year-old woman, under diploma)

"Early on, when I found out I was pregnant, I was stressed about how to change her diaper and other things. I remembered I had a doll; sometimes when no one was home, I would bring it and practice with it to learn how to change diapers." (20-year-old woman, under diploma)

"As soon as I found out I was pregnant, I raised my knowledge wherever I could to give birth to a healthy baby." (16-year-old woman, under diploma)

**Take better care of yourself and your child**. Through a study or a warning from health network experts after pregnancy, some participants realized that they could be at risk for themselves and their children. Therefore, they tried to take care of themselves and their children as much as they could by following the protocols of pregnancy, paying more attention to their health and that of the child, performing screenings, and following the instructions of health experts and doctors.

"When I realized that teenage pregnancy could be dangerous for both me and my baby, I tried to take more care of myself." (21-year-old woman with a diploma)

"From the beginning of my pregnancy, I tried to follow all the instructions given to me by doctors and experts because I knew how important it was for my health and that of my child." (17-year-old woman, under diploma)

"Mostly because I could give birth to a healthy baby, I took care of myself a lot and did all the ultrasounds, and every month I went to our town health center to weigh myself." (16-year-old woman, under diploma)

#### 3.4.2 Negative reactions

This category refers to the negative feelings and reactions adolescent women had after pregnancy and includes the subcategories of trying to kill herself and the kid, fear, and concealment.

**Try to kill herself and the kid**. Early pregnancy puts a lot of stress on adolescent women, especially if the pregnancy happens unintentionally. Some women state that they do not have the conditions for pregnancy and child-rearing at all, so they try to harm themselves and the fetus in a way that would lead to abortion. In more severe cases, due to high stress, even leads to suicide in some participants.

"When the doctor told me for the first time that I was pregnant, I was going crazy. I could not believe it at all. At that moment, I told my husband that I could not raise the baby, and if you wanted me, you would have to help us have an abortion, fortunately, because the fetus had a problem, they agreed to have an abortion." (18-year-old woman with a diploma)

"When I found out I was pregnant, to get rid of the baby, I hit myself on the door and the wall to have an abortion. Sometimes I jumped from a height, and sometimes I hit my stomach hard. It was a terrible day. (21-year-old woman, higher than a diploma)

"My first pregnancy was very difficult for me because I did not know anything and I was far away from my mother to get help. I had a lot of stress. There was so much pressure on me that I once decided to kill myself." (22-year-old woman with a diploma)

**Fear and concealment**. Many participants were frightened after pregnancy. Some of them also denied pregnancy and even hid their pregnancy from their husbands, family, and healthcare systems, which could put their health at greater risk.

"I remember that when my test was positive, I was very scared. I did not know what to do; I was just crying. I could not believe that I was pregnant. I told myself that something must have gone wrong because I was taking pills. I took a pregnancy test twice to make sure I was pregnant. (19-year-old woman with a diploma)

"When I found out I was pregnant, I did not know what to do. I did not know how to tell my husband. I did not tell him anything for two months until he found out himself." (17-year-old woman, under diploma)

"When I got pregnant, I told my husband that he had no right to say anything to anyone. I did not tell anyone; I did not even tell my mother so that no one would understand. I rarely went to parties." (20-year-old woman with a diploma)

"When I got the answer to my pregnancy test, I did not go to our village health center for two months, until they sent me several messages asking why I didn’t go, so I went. Actually, I did not want anyone in the village to know that I was pregnant." (16-year-old woman, under diploma)

"I did not feel good about being pregnant; I always tried to hide from others." (15-year-old woman, under diploma)

"Some teenage women who come to me say, 'Please, no one should know that I'm pregnant.' Some of them even go to hospitals and health centers so rarely that people do not know they are pregnant." (35-year-old midwife with an MA degree)

### 3.5 Consequences

In this study, the consequences of pregnancy in adolescence were analyzed in two categories: destructive and constructive consequences.

#### 3.5.1 Destructive consequences

This category refers to the consequences that adolescent pregnancy can cause for adolescent women or their children and includes the subcategories of child health threat, mother health threat, and inadequate access to health services.

**Threats to the health of the child**. Adolescent women are not yet physically ready for pregnancy, and the pressure and stress that pregnancy imposes on them can endanger the health of their babies. Most participants reported stillbirths, deaths of newborn babies, low birth weight, congenital anomalies, a high risk of sudden infant death syndrome (SIDS), mental retardation, brain injury, and birth injuries.

"The first time I got pregnant, I was 15 years old, but I was lucky that my baby was stillborn; otherwise, I did not know what to do with it." 20-year-old woman with a diploma

"My second child was born at the age of 17, but he had a problem. His lungs were not formed, so he died." (19-year-old woman, under diploma)

"I was very weak myself, so my child was very weak. When she was born, she was so small that I was afraid to hug her." (16-year-old woman, under diploma)

"My daughter has a problem walking. I do not know. Maybe it was because I got pregnant early." (22-year-old woman, higher than diploma)

"My son has a mental problem, and he cannot move well. I curse myself a thousand times a day because one or two people told me that it was because I got pregnant early and did not know how to protect myself properly during pregnancy." 18-year-old woman, under diploma

"Pregnancy in adolescence is dangerous and can endanger the health of children." (55-year-old obstetrician)

**Threat to her mother's health**. Most of the participants stated that they had many problems during pregnancy and that their health was in danger. Some obstetricians and gynecologists have also emphasized that women in the study area are malnourished, which makes pregnancy more difficult. Some of them have been hospitalized frequently during pregnancy or have had a difficult delivery, which eventually led to a cesarean section.

"Women in these areas, who are often malnourished and do farming activities at the same time, face many problems during pregnancy. Most are iron deficient." (31-year-old, BS in obstetrics)

"I was hospitalized about ten times during my nine months of pregnancy, and I told myself a hundred times a day that I was wrong to get married." (17-year-old woman, under diploma)

"During pregnancy and after delivery, my body was very weak, and I felt dizzy all the time." (22-year-old woman, higher than diploma)

"I had a very difficult delivery. First, they told me that I should give birth to my baby naturally. They bothered me all day; finally, they said the baby was not coming, so I had to have a cesarean section." (18-year-old woman, under diploma)

**Inadequate access to health services**. Most of the participants usually learned about their pregnancy late due to their young age and experience, and this endangered their health and that of their children. Of course, part of this late referral could be due to the process of accepting the pregnancy described above, and it took time for them to accept the pregnancy. Also, no health or social organization in the study area is dedicated to pregnant adolescents and provides them with health and social services.

"Some of the women who come to me are several months pregnant, but they are unaware." (55-year-old obstetrician)

"When I found out I was pregnant, no one and nowhere helped me—no education, no counseling, nothing." (15-year-old woman, under diploma)

"Unfortunately, although health centers know that pregnancy in adolescence is dangerous, they do not have any privileges or special attention for pregnant adolescents, and we cannot do anything for them at school because we have no lessons in this field." (29-year-old woman, school counselor)

"Pregnancy in adolescence is associated with many problems and needs more attention, but in our country, it is not important at all, and in many cases, it is encouraged." (32-year-old man, family counselor)

#### 3.5.2 Constructive consequences

This category consists of two sub-categories: increasing support and strengthening the sense of empowerment, which are among the positive consequences that occurred after pregnancy for a limited number of participants.

**Increasing support**: As explained earlier, pregnancy and childbirth are a great advantage for many families in the study area. For this reason, adolescent girls are paid attention to and supported by their families and their husbands' families after pregnancy. Part of this support is to raise awareness among adolescent women about the conditions of pregnancy, and the other part is related to emotional and psychological support. Adolescent women are also more supported during childbirth or childbearing due to their inexperience.

"As soon as my husband's sister found out I was pregnant, she explained everything to me because our age difference is not great and I feel comfortable with her, but she has two children. She got married earlier than me." (19-year-old woman, Illiterate)

"When my mother found out I was pregnant, she brought us food all the time, and she did not let me get bothered." (17-year-old woman, under diploma)

"My elder sister always comforted me and told me not to worry and that I could give birth to a healthy and beautiful baby." (16-year-old woman, under diploma)

**Strengthen the sense of empowerment**. Pregnancy is a critical stage in every woman's life, and she thinks she has reached maturity when she becomes pregnant. Most of the participants in this study stated that they felt better and more empowered after a few weeks of pregnancy and also after the birth of the child. Having a successful pregnancy promotes self-confidence and self-esteem in adolescent women.

"When I found out I was pregnant, I was not so worried that I would not be able to have my baby, but the more it passed, the more I hoped for myself, especially when I went to the doctor and the doctor said my baby was fine." (17-year-old woman with a diploma)

"It felt good to be able to handle my life and give birth to a healthy baby at that young age." (17-year-old woman, under diploma)

"When I saw that I was able to cope with life's problems and give birth to my child, it was the best feeling in the world. I was very good; I felt I could do anything else; I felt empowered." (22-year-old woman, higher than diploma)

## 4 Description of the conceptual model (paradigm)

Based on the results of the analysis of interviews, a conceptual model of the phenomenon of pregnancy in adolescence was formed (Fig. 1). Predisposing conditions for pregnancy in adolescence had social and cultural aspects and were rooted mostly in the culture, customs, and beliefs of the people of the Kurdish regions of Iran, which led to the formation of misconceptions in this field and ultimately strengthened pregnancy behavior in adolescence. Due to the prevalence of pregnancy in adolescence in the study area, adolescent pregnancy had become a custom for women who, if they refused to become pregnant, would face stigma and negative reactions from others. Causal conditions of pregnancy in adolescence were related to the individual on the one hand and the family on the other: a large part of the individual factors were related to a lack of knowledge about contraceptives and the risks of pregnancy in adolescence, and another part was related to improper use of contraceptives. An instrumental look at the birth of a child and considering it as a way to escape from loneliness was another individual factor that caused pregnancy in adolescence. Family factors, consisting of the pressure of the husband and family, which was mostly social, were other causal conditions. Of course, within the structure of families, some women used their pregnancies as a means to commit their husbands to life and to establish their position, which shows the value of pregnancy and childbirth in Kurdish areas because, having children, men work harder for the family than before and try to maintain it. Furthermore, structural factors accelerated the pregnancy process in adolescence through difficult access to contraceptives and the lack of barriers to pregnancy due to unemployment and a lack of education. In the face of pregnancy, women also had two types of positive and negative reactions: some of them prepared for pregnancy, childbirth, and raising children, and others could not cope with this issue and tried to either kill the baby or hide their pregnancy from others and health systems. Eventually, pregnancy in adolescence was associated with positive and destructive consequences, most of which were destructive and endangered the health of the adolescent mother and her baby, but in some cases, due to the social and cultural context of the region and the high value of having a child for adolescent women, they received more support after pregnancy, and their sense of empowerment was strengthened by giving birth to a child.

## 5 Discussion

This study aimed to identify the causes and consequences of adolescent pregnancy among adolescents in Kermanshah, western Iran, based on a grounded theory study. The results have shown that adolescent pregnancy in Iran is affected by various factors, such as individual, family, structural, and socio-cultural factors. The social context of the studied community has led to positive and negative reactions by women towards this issue, and subsequently, this pregnancy is accompanied by negative consequences such as threats to the health of the child, threats to the health of the mother, and improper access to health services. However, considering the social fabric and cultural beliefs related to adolescent pregnancy, it can lead to positive outcomes such as increasing support and strengthening girls’ sense of capability.

Social learning was one of the sociocultural factors underlying pregnancy in adolescents. The prevalence of pregnancy in adolescence in the study area has led to the imitation and learning of pregnancy behavior in other young women. In the study of Ayele et al. (2018), it was reported that girls whose mothers have a history of pregnancy in adolescence are more likely to become pregnant in adolescence [44]. The results of a study by East et al. (2007) showed that adolescents whose mothers or sisters experienced pregnancy in adolescence were more likely to become pregnant in adolescence [45]. Ahinkorah et al.'s (2019) study emphasises the impact of peers on pregnancy in adolescence [46].

Misconceptions about fertility and childbirth were another socio-cultural factor underlying pregnancy in adolescence that was one of the new and significant findings in this study. The root of these misconceptions was a lack of sufficient information on fertility and childbirth. Of course, the existence of religion was also effective in some cases because some women considered delaying pregnancy a sin and tried to get pregnant sooner. Other studies have also shown that religious women tend to have a greater inclination toward pregnancy and consider delaying it a sin [14,47].

Stigma prevention was another social and cultural factor that led to pregnancy in adolescence. This finding is inconsistent with most previous studies because, in other studies, pregnant adolescents were exposed to stigma for having abnormal sex, which led to their isolation [48,49]. This difference is due to social and cultural contexts because, in this study, pregnancy in adolescents was studied in the form of formal marriage, while in other studies, pregnancy may have taken place in the form of extramarital relationships. In the present study, women who delayed pregnancy were exposed to many stigmas, such as the stigma of being infertile, the stigma of laziness, and the stigma of irresponsibility. Therefore, women used to get pregnant to prevent these stigmas. While in other societies, delaying pregnancy after marriage may be common, it is not common in Iran, especially in rural and traditional areas.

Lack of knowledge about how to prevent pregnancy and improper use of contraceptives were two of the individual factors resulting in pregnancy in adolescents. In most previous studies, the non-use of contraceptives has been mentioned as one of the important determinants of pregnancy in adolescents [50]. In this study, part of this lack of awareness and misuse of contraceptives is due to the lack of adequate education on these issues, and a larger part is due to the taboos that make it difficult for families to talk about contraception and instructions for it. These issues lead to unintended pregnancies that put a lot of pressure on adolescent women. In the study of Munakampe et al. (2021), informing adolescents about contraceptives and pregnancy prevention methods has been mentioned as a suggestion to reduce pregnancy in adolescents [51]. In the study of Kassa et al. (2018), the lack of proper communication and parental awareness among adolescents on sexual and fertility issues has been mentioned as one of the determinants of pregnancy in adolescence [12]. Mardi et al. (2018) also reported that one of the main reasons for not using contraceptives among Iranian adolescents is a lack of familiarity with contraceptives [52]. This highlights the need for continuing education before and after marriage for young couples. Premarital education and telephone counseling can be strategies to help women plan for pregnancy and childbirth to prevent their negative consequences.

Inadequate knowledge about the risks of pregnancy in adolescence was another individual determinant of pregnancy that can have social roots because in the Iranian education system, no lesson or program about the risks of pregnancy in adolescence informs adolescents, and families because low awareness cannot pass on the right information to their children. A study by Ahinkorah et al. (2019) reported that adolescents who did not talk to their parents about sex and pregnancy in the family were more likely to become pregnant during adolescence [46]. Part of the reason parents do not give awareness about sexuality to adolescents is because some parents believe that informing adolescents and talking to them about sexuality is sexually arousing and harmful [53]. In a study conducted in Iran, sexual taboos, social concerns about the negative effects of sex education, and an unwillingness to discuss sexual issues were mentioned as the main social and cultural challenges of sexual health education for adolescents [54].

Fear of the side effects of using contraceptives was another interesting finding in this study, which was consistent with previous studies [52,55]. Most women had a negative attitude towards the use of contraceptives and thought that using them could endanger their health and pregnancy in the future. This is due to both poor health literacy, which makes women pessimistic about contraceptives, and men's reluctance to use contraceptives for reasons such as decreased sexual pleasure and anxiety about the consequences.

Another individual factor predisposing to pregnancy in adolescence was filling the vacuum of loneliness. Teenage women became involved in life by giving birth to their children. Of course, this issue was more prevalent among women whose husbands were away from them. The Kurdish regions of Iran are among the most deprived regions of Iran in terms of employment and industry, and for this reason, many men in these regions have to go to work in Tehran or other industrial cities of Iran, because they cannot afford housing and living in those cities, they leave their wives in their hometown and live alone in the city they work in, which creates a sense of loneliness in young women.

One of the important family factors causing pregnancy in adolescence was the pressure of family and husband, which is consistent with the results of previous studies in Iran and other countries [52,56,57]. In most sub-Saharan African countries, adolescent girls may face social pressure to marry and, once married, to have children [58]. Given that adolescent pregnancy in the study area is not only not prohibited but also encouraged, many adolescent women after marriage are pressured to get pregnant by their husbands or husband's family, and this leads to pregnancy. Of course, because in most Kurdish areas, due to poverty and financial problems, young couples are forced to spend the first few years of their lives with the husband's family, this pressure from the husband's family increases and can affect the lives of the young couple. Another part of this pressure can be due to the lack of authority of women to determine the time of pregnancy, as well as the weak power and lack of bargaining of adolescent women in sexual intercourse because the use or non-use of contraceptives is mostly determined by men and women cannot have the right to choose [[59], [60], [61]].

Committing the husband to life and consolidating her position in the family were other family factors that caused pregnancy in adolescence. In most studies, family structure has been mentioned as one of the important determinants of pregnancy in adolescents [29,30,62]. Moridi et al. (2019), in a study conducted among Iranian pregnant adolescents, also reported that adolescent women considered the existence of a child a factor in establishing marital life and felt satisfied with its existence [63].

Intervening conditions for pregnancy in adolescence included structural factors that consisted of two subcategories: no barriers to pregnancy and difficult access to contraceptives. In most studies, higher education and employment have been reported as protective factors against pregnancy in adolescence [[64], [65], [66], [67]]. In this study, since the participating women in most cases did not meet either of these conditions, they entered the pregnancy cycle as soon as possible. It can be said that education and pregnancy in adolescence have a reciprocal relationship: on the one hand, low education leads to pregnancy, and on the other hand, pregnancy in adolescence reduces the possibility of education and, in most cases, leads to adolescents dropping out of school.

Difficult access to contraceptives was another determinant of adolescent pregnancy, which is partly due to the high cost of contraceptives and their poor distribution among different rural areas, but the most important part is the taboos that make it hard not only for women but also for their husbands to buy contraceptives. Previous research showed that access to and use of contraceptives can play an effective role in reducing pregnancy in adolescents [38,68,69]. In a study conducted by Mohammadi Gharghani et al. (2021) among prostitutes in Iran, access restrictions were mentioned as one of the determinants of not using condoms [70].

In the present study, young women in the face of pregnancy had two contrasting types of positive and negative reactions, which is consistent with the research of Sadler et al. (2016 and Moridi et al. (2019) [63]. In a positive reaction, women tried to prepare themselves for raising children and increase their knowledge about the conditions of pregnancy and parenting. They also tried to take better care of themselves and provide safe conditions for themselves and their children as much as possible. However, some other teenage women had a destructive reaction that could endanger the health of both mother and child. They were not ready for pregnancy, and when they heard that they were pregnant, in the first stage they tried to kill themselves or the formed fetus, and in the next stage, they tried to hide the pregnancy from their relatives and even health centers as much as they could. This in itself could put their health at greater risk because pregnant teens need more health care because of the needs they have.

Finally, adolescent pregnancy has positive and negative consequences for women, most of which are negative and can endanger the health of the mother and child. According to the present study, in most previous studies, pregnancy in adolescence has been identified as a threat to the health of mother and child [71,72]. One of the consequences of pregnancy in adolescence is the occurrence of physical problems and childbirth problems. This may be because these women became pregnant at a time when their bodies had not yet fully developed and were not ready to face the stress of sexual intercourse, pregnancy, and childbirth. The incidence of physical problems among women who experience pregnancy in adolescence has also been shown in previous studies [46,73]. Of course, another reason for the occurrence of physical problems in the women under study can be due to low nutritional knowledge as well as economic problems that deprive women of having enough nutrition during pregnancy.

Exposure to high-risk pregnancies and deliveries has been frequently reported by women in the present study, which may be due to pelvic underdevelopment and possibly the coincidence of pregnancy and pubertal growth and development. These consequences have been shown in previous studies [28,71,74]. Onoyase (2020), in a study of Nigerian women, reported that women who experience adolescent pregnancy have many problems during childbirth [75]. In the study of Amjad et al. (2019), low birth weight and adolescent maternal mortality were reported as pregnancy outcomes in adolescence [76]. In a 2014 study by Dehghan-Nayeri and Tajvidi, problems such as hypotension, nausea, backache, headache, stomachache, etc. were reported in pregnant adolescents [77]. Adolescent women in the early days and weeks of marriage have no understanding of the marital relationship and how to prevent pregnancy, so they become pregnant very quickly without wanting to, and because neither their body is ready for such a pregnancy nor their mind is ready to accept such a fact, the process of pregnancy and childbirth becomes difficult and painful for them. This issue can endanger the health of the child and mother so that in some cases it leads to multiple abortions and other problems.

Inadequate access to health services is another consequence of pregnancy in adolescence, which can be due to knowing about their pregnancy late or the time-consuming process of coping with pregnancy, which can endanger the health of adolescent women a lot. Previous studies have reported that pregnant adolescents have less access to health services [78,79]. The study by Kohan et al. (2021) also showed that insufficient access to pregnancy health services, the inability of healthcare workers to provide appropriate services to adolescents, and a lack of counseling services for adolescents are the most important problems facing pregnant adolescents in Iran [80].

In a few cases, pregnancy in adolescence can lead to more support and a stronger sense of empowerment. This finding was consistent with the results of Solivan et al. (2015) because, in their study, adolescent mothers felt more self-efficacy and had the support of their family and sexual partners [81]. In another study, adolescents assessed pregnancy as a positive event that transitions them into adulthood and provides an opportunity for further growth [82]. A 2010 study by Aujoulat et al. reported that many adolescents were happy to be mothers and felt proud [83]. However, regarding the support that adolescent mothers receive, it should be said that the roots of this support go back to social and cultural factors, even though in most studies, pregnancy in adolescence is prohibited by families [84]. In this study, pregnant adolescents were supported more because pregnancy occurred in the form of marriage and family, while in most studies, pregnancy occurred in the form of a relationship outside the marriage.

## 6 Strengths and limitations

This research is one of the few that identifies the causes and consequences of pregnancy in adolescents with the method of grounded theory and can provide comprehensive and useful information to legislators, health workers, and social planners to intervene in reducing pregnancy in adolescence and promoting the health of pregnant adolescents. Another strength of this research is the simultaneous participation of pregnant adolescents and key informants, and since key informants have been selected from a wide range of different and influential people, it has helped a lot to explain the phenomenon. Another strength of the research is the nativeness of the researchers and their long history of conducting research among the study areas, which caused the researchers to be fully acquainted with the customs and traditions of the participants, and this issue helped the researchers gain their trust.

However, this research also has significant limitations. The first and perhaps most important limitation of the study is that it is conducted only among people who have experienced pregnancy in the form of formal marriage and does not include people who have become pregnant outside of marriage. Therefore, it is suggested that future studies focus on both groups. One of the limitations of the study, due to the nature of qualitative research, is the inability to generalize the data and findings to the entire population. Accordingly, this study does not claim to generalize its findings to the entire Kurdish-speaking population of Iran. Generalization would require further studies using quantitative and survey-based approaches to ensure the applicability of the results. Another limitation of the research is that it has been done only among one Iranian ethnic group (the Kurdish ethnic group), which has its own cultural and social context, and the results of the study cannot be generalized to other Iranian ethnic groups or other countries. Therefore, it is suggested that a similar study be conducted among other ethnic groups in Iran and other countries.

## 7 Conclusion

The results showed that adolescent pregnancy in Kermanshah province is influenced by various socio-cultural, family, individual, and structural factors, and women have two types of positive and negative reactions in the face of pregnancy, which can ultimately have positive)such as an increase in support and a strengthening of the sense of empowerment) and negative consequences that, in most cases, are negative and can endanger the health of the adolescent mother and her child.

Therefore, educational and promotional interventions are necessary to prevent pregnancy in adolescents and reduce its negative consequences. Therefore, it is suggested that in the first stage, pregnancy in adolescence should be prevented by correcting the cultural misconceptions of society about pregnancy and childbirth, raising women's awareness about contraceptives and how to use them, and providing easy access to them. Additionally, the results of this study can be shared with healthcare centers to facilitate community-based interventions aimed at reducing teenage pregnancy and mitigating its negative consequences. Also, raising the awareness of women and families about the dangers of pregnancy in adolescence and providing conditions for them to be able to continue their education after marriage can be useful in preventing pregnancy in adolescents. In the next stage, to reduce the negative consequences of pregnancy, measures such as continuous monitoring of maternal and child health, providing free health and psychological counseling for adolescent mothers on how to cope with pregnancy, providing easier access for pregnant women to health and social services, and strengthening social support for them can be taken.

The results of this study can increase the knowledge of policymakers about the reasons for adolescent pregnancy so that they can better prevent it through planning, creating laws, and multi-dimensional interventions. Additionally, by highlighting the negative consequences of adolescent pregnancy and its challenges, they can better make policies to support pregnant adolescents. The Ministry of Health in Iran can collaborate with other ministries such as the Ministry of Youth Affairs and Sports, particularly the Ministry of Education, to address issues related to teenage pregnancy through discussions and activities. One solution is to incorporate a curriculum unit on teenage pregnancy into primary and secondary school textbooks. Furthermore, representing the conditions and challenges faced by pregnant adolescents in the media and social networks is another strategy to raise awareness among the public about the challenges associated with this phenomenon. Moreover, developing health protocols for teenage pregnancy prevention and implementing comprehensive support programs for pregnant adolescents are additional measures that can help mitigate the negative consequences they may face.

## CRediT authorship contribution statement

**Javad Yoosefi Lebni:** Writing – review & editing, Formal Analysis, Data curation. **Ahmad Ahmadi:** Writing – original draft, Formal analysis. **Seyed Fahim Irandoost:** Writing – review & editing, Formal analysis, Conceptualization. **Mandana Saki:** Writing – review & editing, Conceptualization. **Hossein Safari:** Writing – original draft, Investigation, Data curation. **Nafiul Mehedi:** Writing – review & editing, Formal analysis.

## Data availability statement

The data that support the findings of this study are available from the 10.13039/100012021Iran University of Medical Sciences, but restrictions apply to the availability of these data, which were used under license for the current study and so are not publicly available. The data are, however, available from the corresponding authors on reasonable request and with the permission of the Iran University of Medical Sciences.

## Ethical approval

The study was approved by the Ethics Committee of Iran University of Medical Sciences (Code: IR.IUMS.REC.1400.1215). Informed written consent was obtained both for participation in the study and for audio recording. In addition, the researchers obtained written consent from the spouses of the participants who were under 18 years of age.

## Funding

This research did not receive any specific grant from funding agencies in the public, commercial, or not for profit sectors.

## Declaration of competing interest

The authors declare that they have no known competing financial interests or personal relationships that could have appeared to influence the work reported in this paper.
